# Is antibiotic prophylaxis necessary in mid-urethral sling surgery?

**DOI:** 10.1007/s00192-020-04408-z

**Published:** 2020-07-02

**Authors:** Rune Svenningsen, Sigurd Kulseng-Hanssen, Ellen Bettina Casanova Kråkenes, Hjalmar August Schiøtz

**Affiliations:** 1 Department of Gynecology, Oslo University Hospital, Ullevål, PO Box 4956 Nydalen, 0424 Oslo, Norway; 2 Faculty of Medicine, University of Oslo, Oslo, Norway; 3 The Norwegian Female Incontinence Registry, Oslo University Hospital, Ullevål, Oslo, Norway; 4 Department of Obstetrics and Gynecology, Haukeland University Hospital, Bergen, Norway; 5 Department of Obstetrics and Gynecology, Vestfold Hospital Trust, Tønsberg, Norway

**Keywords:** Antibiotic prophylaxis, Mid-urethral slings

## Abstract

**Introduction and hypothesis:**

Antibiotic prophylaxis is commonly used when implanting a mid-urethral sling (MUS) for female stress urinary incontinence. Use of antibiotics may lead to adverse events and the development of antibiotic resistance. This study compared a variety of outcomes after MUS surgery with and without antibiotic prophylaxis using data from the national Norwegian Female Incontinence Registry.

**Methods:**

Preoperative and 6–12 months postoperative subjective and objective data from 28,687 patients who received MUS surgery from 1998 through 2017 were extracted from the registry. Categorical outcomes were compared between women with or without antibiotic prophylaxis using chi-square test for independence. Primary outcome was incidence of postoperative surgical site infection (SSI). Secondary outcomes were incidence of tape exposure, de novo or persistent urgency urinary incontinence, postoperative pain > 3 months, subjective and objective cure rates, and patient satisfaction.

**Results:**

Antibiotic prophylaxis was used in 9131 and not used in 19,556 patients. SSIs and prolonged postoperative pain occurred significantly more often without antibiotic prophylaxis. Subjective and objective cure rates were significantly higher and tape exposures significantly lower in women not receiving prophylactic antibiotics. There were no significant differences in other outcomes.

**Conclusions:**

Antibiotic prophylaxis resulted in fewer women developing postoperative infections or prolonged postoperative pain after MUS surgery, but did not offer protection against tape exposure. The differences in cure rates were small and probably without clinical relevance. If a small increase in surgical site infections is accepted, the routine use of antibiotic prophylaxis can probably be omitted.

## Introduction

Tension-free mid-urethral slings (MUS) are frequently used in the surgical treatment of female stress urinary incontinence (SUI) and mixed urinary incontinence (MUI) and are currently considered the gold standard [1]. In these procedures a narrow polypropylene mesh tape is introduced through a small vaginal incision to create a tension-free support for the urethra. The MUS procedures have good subjective and objective efficacy for both SUI and stress dominant MUI and are considered safe with few short- or long-term serious complications [1–3].

It is generally accepted that antibiotic prophylaxis should be used during implant surgery, and it is also recommended in clean-contaminated surgery according to the 2019 guidelines from the National Institute for Health and Care Excellence (NICE) [4]. Regardless of preoperative vaginal cleansing procedures, it is not possible to obtain a sterile operative field in the vagina prior to vaginal surgery, and the vagina is therefore considered a clean-contaminated field [4]. As the MUS procedures involve inserting a permanent implant through a clean-contaminated field, antibiotic prophylaxis is commonly used in connection with these operations.

However, in addition to the economic cost of using antibiotics, there is a risk of adverse events occurring, such as allergic reactions, fungal infections and *Clostridium difficile* colitis as well as the development of antibiotic resistance in the population. Therefore, some hospital departments prefer not to use antibiotic prophylaxis when performing MUS surgery.

In Norway, nearly all female incontinence operations are carried out in gynecological departments in public hospitals under standard protocols, usually as day surgery. An unpublished survey performed by the senior author (Schiøtz) in December 2018 revealed that some Norwegian departments offering MUS procedures never use antibiotic prophylaxis while some always do. A few departments reported using antibiotic prophylaxis in high-risk cases only.

The Norwegian Female Incontinence Registry was established in 1998 by a group of dedicated urogynecologists to evaluate the outcomes of incontinence surgery in Norway [5]. The registry has over the years evolved into a compulsory, nationwide registry funded by the Health Authorities. All public hospitals in Norway performing incontinence surgery in women are under obligation to report to the registry their pre- and postoperative subjective and objective data as well as the type of procedure and any complications. The last report from the registry (2018) showed that 99.4% of all procedures performed were reported to the registry. Annual reports from the registry indicate that the overall incidence of surgical site infections (SSI) is low (< 1%), but potential differences in infection rates between hospitals which routinely use antibiotic prophylaxis and those that do not have never been evaluated. This study therefore aimed to compare the incidence of postoperative SSIs after MUS surgery in women who were or were not given antibiotic prophylaxis at the time of surgery.

It has been suggested that some patients develop a chronic tape-related inflammatory tissue response or a chronic low-grade infection in biofilm along the tape that remains subclinical and undetected, but this might cause other problems such as tape exposure, de novo urgency urinary incontinence (UUI), persistent bothersome urgency symptoms and chronic pain in some cases requiring tape removal [6, 7]. We hypothesized that the risk of these negative outcomes would be higher in the group without antibiotic prophylaxis. This study’s secondary aim was therefore to also evaluate the incidence of tape exposure, de novo and persistent UUI, prolonged postoperative pain and potential differences in subjective and objective cure rates and treatment satisfaction in women with or without antibiotic prophylaxis.

## Materials and methods

**Fig. 1:**
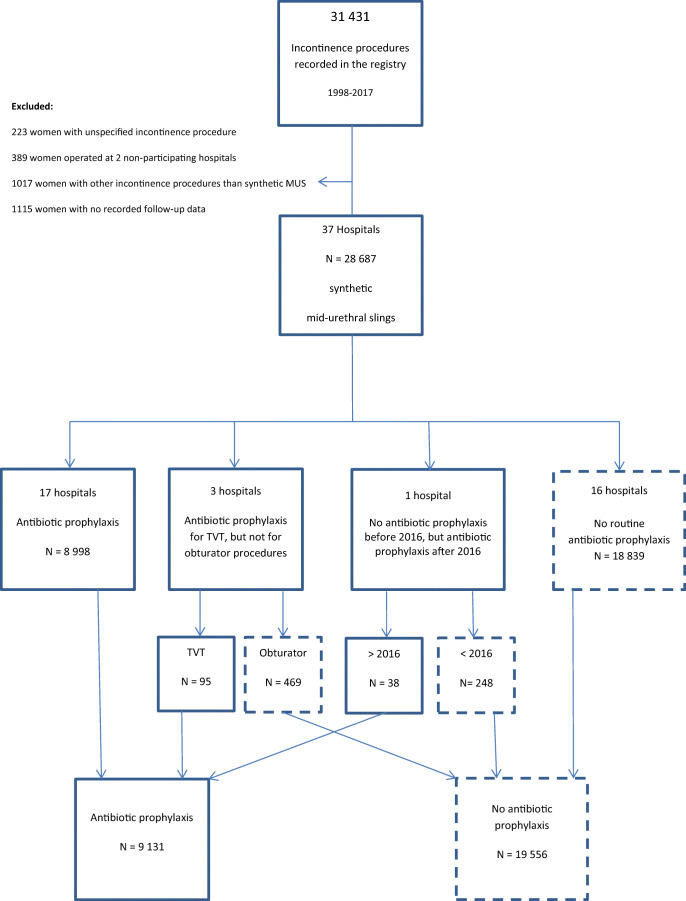
Flowchart of included patients

This registry-based cohort study used data stored in the compulsory national Norwegian Female Incontinence Registry from all women who had undergone synthetic mid-urethral sling (MUS) surgery for stress urinary incontinence (SUI) or stress-dominant mixed urinary incontinence (MUI) in the period 1998 (inception of the registry) through 2017 with recorded follow-up data through 2018. Data extraction from the registry was done in January 2020. Retropubic tapes, transobturator tapes and minislings were all included. The extracted data were stratified based on the use of antibiotic prophylaxis. As the use of antibiotics are not routinely reported to the registry, this stratification was based on a survey performed in December 2018 in which the local doctor responsible for reporting data to the registry at all reporting hospitals was asked about their department’s guidelines for the use of antibiotic prophylaxis during MUS surgery. At the time of data extraction there were 38 reporting public hospitals and 1 reporting private hospital. The private hospital and one public hospital were excluded from the study because of not participating in the survey (private hospital) or not having guidelines for antibiotic prophylaxis use (public hospital). Beyond that, only women with no recorded follow-up data were excluded (Fig. 1). Five hospitals which did not use antibiotic prophylaxis routinely used prophylaxis in patients perceived to be at high risk of infection, mainly those with type I diabetes mellitus. Accurate data on antibiotic use for these women were not obtainable, but were estimated to constitute < 5% and therefore have little impact on the analyses [8]. Women from these hospitals were therefore added to the non-prophylaxis group. The routine for prophylactic antibiotic treatment was a single dose of a first- or second-generation cephalosporin with or without metronidazole given in connection with the procedure.

In Norway, the evaluation of women before any incontinence operation includes a questionnaire validated in Norwegian for subjective data as well as a standardized cough/jump pad-weighing stress test for objective data, which has been found to be highly reproducible [9, 10]. The same questionnaire and cough/jump stress test are also used at the mandatory 6–12-month follow-up and at any subsequent visits. A stress urinary incontinence index score (range 0–12) and an urgency urinary incontinence index score (range 0–8) are calculated from the questionnaire to evaluate the degree of stress or urgency symptom bother [9]. A higher score signifies more symptoms, and 0 indicates no symptoms. At follow-up, the women also answer a question on treatment satisfaction with five choices ranging from “very satisfied” to “very dissatisfied.” The cough/jump pad-weighing stress test consists of 20 jumping jacks and three forceful coughs in the standing position with 300 ml bladder volume [10]. Visible leakage or an increase in pad weight ≥ 1 g constitutes a positive test. The completed questionnaire and the results from the cough/jump pad-weighing stress tests as well as any urodynamic data, type of procedure and any surgical complications during or after surgery are reported consecutively to the registry in deidentified form.

The primary outcome of this study was the incidence of the total number of postoperative surgical site infections (SSI) registered at any time after surgery. SSIs are recorded as either superficial or deep in the registry. A subgroup analysis was therefore also performed on the women registered with deep (more severe) infections. Women registered with both deep and superficial SSIs were counted as deep. As the majority of urinary tract infections (UTIs) are treated outside the hospital at the general practitioner’s office and rarely reported to the registry, UTIs were not included as an outcome for this study. The secondary outcomes were as follows: (1) incidence of vaginal tape exposure (both symptomatic and asymptomatic), (2) incidence of persistent UUI, defined as urgency urinary incontinence index score ≥ 2 before and after surgery, (3) incidence of de novo UUI defined as a postoperative urgency urinary incontinence index score ≥ 2 among those with a preoperative score of 0, (4) subjective cure rate defined as a postoperative stress urinary incontinence index score ≤ 2, (5) objective cure rate defined as 0 g leakage on the postoperative cough/jump pad-weighing stress test, (6) patient satisfaction defined as the percentage of women answering “very satisfied” with treatment when given the choices “very satisfied,” “moderately satisfied,” “neither satisfied nor dissatisfied,” “moderately dissatisfied” and “very dissatisfied” and (7) prolonged postoperative pain defined as pain > 3 months following surgery. All secondary outcomes were calculated from the 6–12-month follow-up data except for the vaginal tape exposure data in which recorded cases at any time after surgery were counted. Furthermore, we calculated the patient number needed to treat (NNT) with antibiotic prophylaxis to avoid one case of postoperative SSI.

The study was approved by the Regional Committee for Medical and Health Research Ethics of the South-East of Norway (reference no. 28964) as well as the Department Head and Institutional Personal Data Officer at Oslo University Hospital (OUS) having the legal responsibility for the Registry. All women had previously signed a consent form allowing the storage of data in the registry for quality assurance measures. Since only anonymous data from the registry were used for the present study, obtaining additional patient consent for the present study was not deemed necessary.

Statistical analyses were done using Statistical Package for the Social Sciences (SPSS-PC), version 25. Differences in categorical data were tested using chi-square test for independence. The analyses for each outcome were done as per protocol in which women with missing data were removed from the denominator. A significance level of 5% was used. Due to the large number of women in the registry, no sample size calculations were needed.

## Results

At the time of data extraction, a total of 31,431 women were recorded as having had some form of incontinence surgery in the study period, among whom 28,687 matched the inclusion criteria; see the flowchart in Fig. 1. Antibiotic prophylaxis was used in 9131 (32%) and not used in 19,556 (68%) patients. The results for the primary and secondary outcomes are presented in Table 1. Surgical site infection (SSI), both total number and deep (severe), occurred significantly more frequently in the women not given antibiotic prophylaxis (total: 1.2% vs. 0.6%, *p* < 0.01, deep: 0.5% vs. 0.2%, p < 0.01; Table 1). The same was demonstrated for prolonged postoperative pain > 3 months (no prophylaxis: 0.6% vs. prophylaxis: 0.4%, *p* = 0.01); Table 1). There were no significant differences between groups in the incidence of de novo or persistent urgency urinary incontinence or treatment satisfaction. However, subjective and objective cure rates were statistically significantly higher in women not given antibiotic prophylaxis, but the clinical differences were small (subjective: 79.5% vs. 76.6%, *p* < 0.01 and objective: 90.8% vs. 87.5%, *p* < 0.01; Table 1). Tape exposure was lower in the no antibiotic prophylaxis group (1.1% vs. 1.6%, *p* < 0.01; Table 1). The number needed to treat to avoid one SSI was calculated at 166 and for deep (severe) infections at 333.

**Table 1:** Results from the 6–12-month follow-up

Outcomes	Antibiotics *N* = 9131		No antibiotics *N* = 19,556		*P* value
	Cases/total	Percentage	Cases/total	Percentage	
Surgical site infection (total)	51/9131	0.6	231/19,556	1.2	< 0.01
Surgical site infection (deep)	14/9131	0.2	95/19,556	0.5	< 0.01
Tape exposure	150/9131	1.6	221/19,556	1.1	< 0.01
De novo UUI^a^	224/1590	14.1	573/3865	14.8	0.48
Persistent UUI^b^	3382/6659	50.8	7186/14,012	51.3	0.51
Subjective cure rate of SUI^c^	6249/8159	76.6	14,092/17,726	79.5	< 0.01
Objective cure rate of SUI^d^	5569/6364	87.5	13,039/14,367	90.8	< 0.01
Very satisfied with treatment	6992/8423	83.0	15,257/18,297	83.4	0.45
Postoperative pain > 3 months	37/9131	0.4	125/19,556	0.6	0.01

^a^Postoperative urgency urinary incontinence (UUI) index score ≥ 2, when having a preoperative score of 0

^b^Urgency urinary incontinence (UUI) index score ≥ 2 before and after surgery

^c^Postoperative stress urinary incontinence (SUI) index score ≤ 2

^d^0 g leakage on the postoperative cough/jump pad-weighing stress test

## Discussion

The postoperative surgical site infection (SSI) rate in this study was significantly higher among women who did not receive antibiotic prophylaxis. However, even without such prophylactic treatment the incidence was low at 1.2%, and the number needed to treat with antibiotics to prevent one SSI was high at 166. A subgroup analysis on the even rarer, but potentially more serious deep infections also showed a significant difference, but the NNT was here even higher at 333.

The incidence of prolonged postoperative pain was statistically significantly higher among the women not receiving antibiotic prophylaxis (0.6% vs. 0.4%), but prolonged postoperative pain was rare in both groups. We found no overlap between women registered as having prolonged pain and the women registered with SSI; data not shown. These numbers are reassuring for hospitals and surgeons who prefer to carry out mid-urethral sling (MUS) procedures without giving antibiotic prophylaxis. Internationally, the reported incidence of SSI after MUS was 0.6–0.7% in the 2017 Cochrane systematic review [1]. However, how frequently antibiotic prophylaxis was employed in the studies evaluated in that review is not known. Our results are also in line with a recent publication from Japan showing a total incidence of SSI as low as 0.22%, but also in this study the use of antibiotic prophylaxis varied widely among hospitals [11]. The patients in our study who received prophylactic antibiotics had fewer SSIs than both the 5% estimated by NICE in 2019 for SSIs in general [4] and the 5.5% given in a 2008 review by Stanford and co-workers encompassing all types of MUS [12]. Tension-free mid-urethral slings have been used for > 20 years, and the reported complication rates have been low [1]. Surgical treatment is, however, never completely without risk, and SSIs after implant surgery carry the risk of serious morbidity and even mortality [12]. Postoperative SSI also increases treatment costs and may compromise the surgical result. The use of a first- or second-generation cephalosporin carries a small risk of serious adverse drug reactions, such as allergic reactions and *Clostridium difficile*-associated diarrhea [13]. However, when used as a single perioperative dose, the environmental impact with development of antibiotic resistance is very low [14].

When a medical device such as a polypropylene mesh is implanted into the body, a complex and extensive series of foreign body reactions occurs, usually ending with the implant being accepted by the tissues and covered with a fibrous capsule [15–17]. The presence of a foreign body may further induce local immunosuppression mediated through cytokines and thus improve the chance of survival of any bacteria near the foreign body [18]. Bacteria rapidly form a biofilm on the surface of the foreign body, and bacteria in biofilm can be difficult to eradicate with antibiotic treatment as they multiply slowly and are protected against antibiotics due to enhanced antibiotic tolerance and resistance [19–21]. The formation of biofilm may be an explanation for the small increase in the incidence of prolonged postoperative pain found in our study as an association between biofilm and pain has also been demonstrated by others evaluating prolonged pain after temporary or permanent synthetic implant placement [22, 23]. However, in actual numbers the incidence with or without antibiotic prophylaxis was low at 0.4% and 0.6%, respectively. Unfortunately, our data did not contain information on the number of women needing a partial or total revision of the implant because of prolonged pain.

MUS tape exposure is often asymptomatic, and the diagnosis may be missed unless a vaginal examination is carried out. In our study we found that tape exposure was noted significantly more often among women in the antibiotic prophylaxis group. We are unable to propose a biologically plausible explanation for why antibiotic prophylaxis might increase the risk of impaired wound healing. We therefore believe that this is a spurious finding and that the explanation is more likely that some of the operating departments have not diagnosed all their cases of asymptomatic tape exposure, while others have carried out postoperative vaginal inspection more frequently. Except for the finding of a slight reduction in the incidence of prolonged postoperative pain in the antibiotic prophylaxis group, there is little else in our results that supports the hypothesis that chronic inflammation or low-grade infection in biofilm along the implant results in more women developing pain, vaginal wound dehiscence or persistent or de novo urgency urinary incontinence as has been suggested by others [6, 7].

The major strength of this study is the use of data from a large, compulsory national registry with nearly 100% completeness that encompasses all types of patients having been operated on by surgeons with different levels of experience in all regions of the country, minimizing the risk of selection bias that is often inherent in prospective, comparative trials. The study therefore has a high degree of external validity. The high number of patients also facilitates the use of robust statistics.

As with all registry studies there are inherent weaknesses. It is inevitable that some complications are not reported correctly to the registry, and, as previously mentioned, we believe this applies to the reporting of tape exposure. If several departments with the same routines for using antibiotic prophylaxis consistently neglect to report certain complications, this could skew the results and cause a bias. However, we have no reason to believe that the non-reporting of infection was biased in this study; any underreporting would most likely be distributed equally among the groups. Regretfully, we could not evaluate the effect of antibiotic prophylaxis on postoperative UTIs. The incidence of postoperative UTIs recorded in the registry was only 0.4% (data not shown). As others have demonstrated rates of postoperative UTIs as high as 34% [24], we believe this is due to a systematic underreporting, as treatment is most likely being given at the general practitioners office. It might also be considered a weakness that it was only possible to analyze the variables that are routinely reported to the registry, and any adjustment according to body mass index, menopausal status, medications, etc., was therefore not possible. Additionally, using registry data always entails the risk of missing data or inaccuracies in the individual entries, which may potentially impact results. Furthermore, we cannot completely rule out the possibility that the local guidelines regarding antibiotic prophylaxis were not always followed. Some high-risk patients may have received antibiotics in breach of local guidelines while in other cases prophylaxis was not used for reasons not given. In this analysis, we made the assumption that the total number of protocol breaches was small and equally distributed among women receiving or not receiving antibiotics.

In conclusion, in this study the total incidence of postoperative surgical site infections and the incidence of deep (severe) infections after mid-urethral sling procedures were significantly higher when prophylactic antibiotics were not used. However, in absolute numbers the SSIs were rare with a high NNT. In the secondary outcomes we found an increased risk of prolonged postoperative pain without antibiotic prophylaxis, but the incidence was very low in both groups. In all other secondary outcomes we found no consistent differences of clinical importance, but we note that antibiotic prophylaxis does not seem to offer protection against tape exposure or persistent or de novo urgency urinary incontinence. Consequently, we conclude that if one accepts the small increase in surgical site infections, the routine use of antibiotic prophylaxis can probably be omitted.
